# Absence of Pupillary Reflexes in Pediatric Acute Liver Failure and Neurological Outcome After Liver Transplantation

**DOI:** 10.1111/petr.70076

**Published:** 2025-04-10

**Authors:** Kirsten J. Schouwstra, René Scheenstra, Ruben H. de Kleine, Vincent E. de Meijer, Sander T. H. Bontemps, Henkjan J. Verkade, Deborah A. Sival

**Affiliations:** ^1^ Pediatric Neurology, Department of Neurology University of Groningen, University Medical Center Groningen Groningen the Netherlands; ^2^ Pediatric Gastroenterology/Hepatology, Department of Pediatrics University of Groningen, University Medical Center Groningen Groningen the Netherlands; ^3^ Hepatobiliary Surgery and Liver Transplantation, Department of Surgery University of Groningen, University Medical Center Groningen Groningen the Netherlands; ^4^ Pediatric Intensive Care, Department of Pediatrics University of Groningen, University Medical Center Groningen Groningen the Netherlands

**Keywords:** brain herniation, brain stem reflexes, neurological outcome, pediatric acute liver failure (PALF), pupillary reflexes, radiological imaging

## Abstract

**Background:**

Pediatric Acute Liver Failure (PALF) frequently requires liver transplantation (LTx). The neurological condition can deteriorate rapidly, but the difficulty in assessing the (ir)reversibility of neurological symptoms can hamper therapeutic decision‐making, including transplantation. We aimed to determine the association between pupillary reflexes (PR), brain stem reflexes (BSR), radiological signs of brain herniation, and subsequent neurological outcome.

**Methods:**

We analyzed a retrospective, observational cohort of PALF patients with severe hepatic encephalopathy (grade III–IV), admitted to our national pediatric liver transplantation center between 1993 and 2023. We subdivided the patients into groups with PR present or PR absent. We compared the two groups for pre‐treatment neurological and neuro‐radiological parameters and related the findings to neurological outcomes.

**Results:**

Survival rate in patients with PR present was higher compared to patients with PR absent [70% (26/37) and 29% (4/14); resp., *p* = 0.008]. In the absence of PR, neurological outcome could still be favorable after LTx (*n* = 3/6). Presence or absence of BSR was not related to the outcome in terms of survival or death. Radiologically proven brain herniation was associated with mortality (6/7) or minimally conscious state (1/7), irrespective of undergoing a LTx or not.

**Conclusions:**

Although absence of PR is associated with a poor prognosis, the neurological outcome can still be favorable after LTx. Radiological signs of brain herniation are strongly associated with mortality or severe neurological outcomes, irrespective of subsequent transplantation. We therefore advocate that absence of PR should be an indication for radiological imaging to assess brain herniation before making major treatment decisions.

## Graphical abstract

We analyzed a retrospective, observational cohort of 51 patients with pediatric acute liver failure with severe hepatic encephalopathy (grade III‐IV), admitted between 1993 and 2023. The survival rate in patients with present PR was higher compared to patients with absent PR (70% and 29%, respectively). In the absence of PR, the neurological outcome could still be favorable after liver transplantation (LTx) (*n* = 3/6). Radiologically proven brain herniation was associated with mortality (6/7) or minimally conscious state (1/7), irrespective of undergoing a LTx or not.

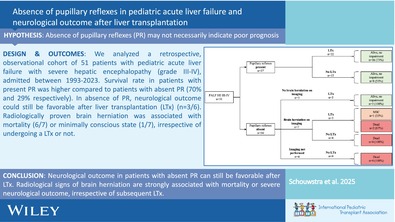

**Abbreviations**
BSR: brain stem reflexes
CR: corneal reflex
HE: hepatic encephalopathy
LTx: liver transplantation
MCS: minimally conscious state
OCR: oculocephalic reflex
PALF: pediatric acute liver failure
PR: pupillary reflex

## 1 Introduction

Pediatric acute liver failure (PALF) is a rare, life‐threatening condition. The underlying etiology varies according to age and includes metabolic diseases, drug toxicity, and viral infections, but in up to 50% no underlying cause can be identified [1, 2, 3]. PALF usually presents in children without pre‐existing liver disease [4, 5], and is characterized by a rapidly developing hepatocellular injury and loss of major hepatic functions, such as protein synthesis, detoxification, and/or biliary secretion [5, 6]. PALF can recover either spontaneously or upon symptomatic or targeted treatment such as liver transplantation (LTx). However, PALF can also deteriorate rapidly, leading to severe complications of absent liver functions, such as multiorgan failure, neurological symptoms, brain herniation, and death. Accordingly, PALF has been an accepted indication for urgent LTx.

During the evolution of PALF, repeated neurological examinations can provide helpful information on the progression of the disease and, if no donor organ has become available, on the chance that PALF has progressed to irreversible neurological consequences, which may make transplantation no longer justified. The neurological symptoms associated with PALF are initially reversible but in the absence of spontaneous recovery or timely LTx, they eventually become irreversible. These symptoms are the result of a complex interaction of various events including accumulation of neurotoxins such as ammonia, damaged blood‐brain barrier, systemic pro‐inflammatory response, loss of cerebral autoregulation, and activation of microglia and astrocytes leading to neuro‐inflammation [7, 8].

Frequently, PALF is associated with progressive hepatic encephalopathy (HE) and, upon further progression of PALF, with the development of cerebral oedema, brain herniation, irreversible coma, and death [1, 4, 9, 10, 11, 12, 13]. HE is clinically graded from grade I–IV, according to the West Haven Criteria [14]. The risk of cerebral edema increases with the severity of HE, varying from 25% to 35% in grade III to more than 65% in grade IV HE [15]. Children with HE grade III show symptoms of confusion and somnolence but do respond to verbal stimuli, whereas children with HE grade IV are in a state of coma or stupor and do no longer respond to stimuli [16]. Whenever possible, treatment aims to cure the underlying factors leading to PALF, but frequently this is impossible or not sufficiently effective, leaving supportive measures as the only option. Although LTx can improve the survival rate (> 75%), a suitable donor liver is not always available in time [4, 17, 18, 19].

In children with PALF and HE grade III–IV, repeated examination of pupillary reflexes (PR) and brain stem reflexes (BSR) is an important parameter for the assessment of the neurological condition and its progression over time. These reflexes can be used for coma assessment in the presence of brain edema and/or brain herniation. So far, it has remained unclear to what extent absent PR relates to irreversibility of neurological damage or outcome. We hypothesized that the absence of PR in PALF may not necessarily indicate a poor prognosis. To address the importance of PR on ultimate neurological outcome during the evolution of PALF, we performed a retrospective observational cohort study of patients with PALF admitted to our national pediatric liver transplant center over the last 30 years. We also evaluated the additional value of brain stem reflexes (BSR) and brain imaging (assessment of brain herniation) in relation to the neurological outcome.

## 2 Patients and Methods

### 2.1 Study Design

This retrospective observational cohort study was performed at the Beatrix Children's Hospital of the University Medical Center Groningen, the single national pediatric liver transplantation center of the Netherlands. We performed a retrospective analysis of data extracted from the medical files of all consecutive patients diagnosed with PALF. The study is approved by the Institutional Review Board of the University Medical Center Groningen (UMCG, 202000140).

### 2.2 Patients

The study cohort consisted of patients with PALF and HE grade III or IV, who were admitted between 1993 and 2023. We retrieved patients by searching the hospital registry for PALF as the final diagnosis (*n* = 123) and subsequently excluded patients with HE grade ≤ 2 (*n* = 75), resulting in 52 patients fulfilling the inclusion criteria. Parent(s)/caregiver(s) of the patients received an information letter regarding our intent to process patient data in an anonymous way, and informed consent for inclusion in the study was obtained in 51/52 patients.

### 2.3 Parameters

The various parameters were obtained, either the data set on the day of availability of a donor organ (in case of LTx) or, in case of no LTx, the data sets at peak value. The timing of outcome data inclusion was the last neurological and laboratory data before discharge from the hospital. In each patient, we compared the neurological and laboratory data during PALF and before discharge.

#### 2.3.1 Neurological Parameters (Used for Coma Assessment)

In children with PALF and HE III–IV, neurological assessment was performed by a neurological consultant and consisted of determining PR and BSR (consisting of oculocephalic reflex (OCR) and corneal reflex (CR)). The PR is an essential indicator of the autonomic nervous system, and the absence of PR may indicate strongly increased intracranial pressure (uni‐ or bilateral, for instance due to brain herniation) and/or neurotoxicity (bilateral). Either bi‐ or unilateral absence of OCR and CR may provide information about the integrity of brainstem function, which is crucial for vital functioning [20]. Both unilateral and bilateral absence of PR and BSR were categorized as absent.

### 2.4 Characterization of Radiological Indications for Brain Herniation

All MRIs and CTs were assessed by pediatric neuroradiologists. We subdivided all radiological scans according to the presence or absence of brain herniation and to the type of brain herniation: uncal herniation and/or transtentorial herniation.

### 2.5 Statistical Analysis

Statistical analysis was performed using SPSS statistics 26 for Windows. The normality of the distribution of quantitative data was assessed with the Shapiro–Wilk Test. The neurological outcome data between the study and control groups were analyzed using the Mann Whitney U test. We used the Chi‐Square test to analyze the categorical neurological parameters. All statistical tests were two‐sided, and *p*‐values of < 0.05 were considered statistically significant.

## 3 Results

Between 1993 and 2023, we identified 52 patients fulfilling the inclusion criteria of PALF and HE grade III–IV. We excluded one patient because of lacking informed consent. Table 1 shows the baseline characteristics.

**TABLE 1:** Patient characteristics and pre‐treatment liver function parameters.

	Study group	Present PR	Absent PR	*p*
Patients, *n* (%)	51	37 (70)	14 (30)	
Age median (range)	3 (0–16)	3 (0–16)	4 (0–15)	0.468
Sex: male/female	29/22	21/16	8/6	0.978
Laboratory data (range)				
INR	6.8 (2.9–10)	6.1 (2.9–10)	8.7 (3.6–10)	0.338
Bilirubin total	348 (31–805)	348 (31–805)	359 (70–730)	0.816
Ammonia	176 (74–529)	161 (74–529)	207 (80–307)	0.530
Etiology PALF, *n* (%)				
Unknown	25 (49)	15 (41)	10 (71)	
Wilson disease	4 (8)	4 (10)	0 (0)	
Infectious	7 (13)	5 (14)	2 (14)	
GALD	4 (8)	4 (10)	0 (0)	
Intoxication	3 (6)	3 (8)	0 (0)	
Autoimmune hepatitis	2 (4)	2 (5)	0 (0)	
Mitochondrial disease	1 (2)	1 (3)	0 (0)	
Post‐surgery	1 (2)	1 (3)	0 (0)	
Metabolic disorder	2 (4)	1 (3)	1 (7)	
Leukemia	1 (2)	0 (0)	1 (7)	
Veno‐occlusive disease	1 (2)	1 (3)	0 (0)	

*Note:* Patient characteristics and pre‐treatment liver function parameters. Laboratory data represent data sets at the availability of a donor organ (in case a LTx was performed) or, in case of no LTx, the data sets at peak value. Outcomes are indicated as median values per group (range).

Abbreviations: INR, international normalized ratio; LTx, liver transplantation.

### 3.1 Subdivision Study Group

Children with PALF grade III–IV (*n* = 51) were assigned to the neurological subgroup with PR either present (*n* = 37) or absent (*n* = 14). The survival rate was significantly higher in the subgroup with PR present compared to the subgroup with PR absent; 70% (26/37) and 29% (4/14), respectively; *p* = 0.008. For causes of deaths, see Table 2. In the following paragraphs, we provide detailed results per subgroup.

**TABLE 2:** Causes of death in study group.

Case number	Etiology PALF	PR	HE III‐IV	LTx	Dead	Cause of death
1	Infectious	+	T = 0	T = 1	T = 4	Multiorgan failure
2	Unknown	+	T = 0	T = 3	T = 44	Unknown
3	Unknown	+	T = 0	T = 3	T = 29	IB and peritonitis
4	POLG‐disease^a^	+	T = 0	T = 4	T = 69	Status epilepticus
5	Unknown	+	T = 0	T = 4	T = 94	Renal insufficiency and BMF
6	GALD	+	T = 0	n/a	T = 4	Multiorgan failure
7	Unknown	+	T = 0	n/a	T = 3	Intracerebral hemorrhage
8	GALD	+	T = 0	n/a	T = 20	Multiorgan failure
9	GALD	+	T = 0	n/a	T = 9	Intracerebral hemorrhage
10	GALD	+	T = 0	n/a	T = 7	Multiorgan failure
11	VOD	+	T = 0	n/a	T = 20	Multiorgan failure
12	Infectious	−	T = 0	n/a	T = 1	Multiorgan failure
13	Intoxication	−	T = 0	n/a	T = 2	Brain herniation
14	Leukemia	−	T = 0	n/a	T = 2	Brain herniation
15	Infectious	−	T = 0	n/a	T = 1	Brain herniation
16	Unknown	−	T = 0	T = 2	T = 5	Brain herniation
17	Unknown	−	T = 0	T = 1	T = 3	Brain herniation
18	Unknown	−	T = 0	n/a	T = 3	Brain herniation
19	Unknown	−	T = 0	n/a	T = 2	Multiorgan failure
20	Unknown	−	T = 0	n/a	T = 2	Unknown
21	Unknown	−	T = 0	n/a	T = 2	Multiorgan failure

*Note:* Causes of death in study group.

Abbreviations: −, absent; +, present; BMF, bone marrow failure; GALD, gestational alloimmune liver disease; HE, hepatic encephalopathy; IB, intestinal bleeding; LTx, liver transplantation; n/a, not applicable; PALF, pediatric acute liver failure; PR, pupillary reflexes; T, time in days after HE grade III‐IV; VOD, veno‐occlusive disease.

^a^ Diagnosed after LTx.

#### 3.1.1 PALF Grade III‐IV Patients With Pupillary Reflexes Present

**FIGURE 1:**
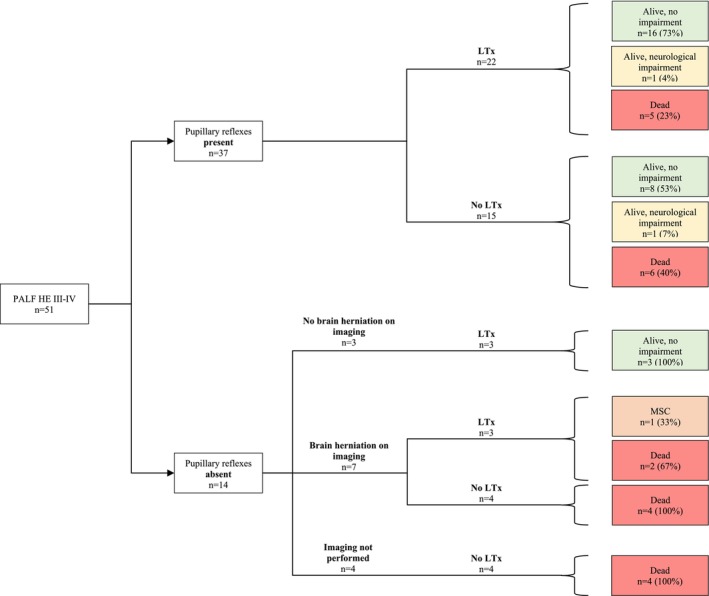
Flow diagram representing the outcome in children with PALF HE III‐IV. The study group was subdivided based on the presence and absence of pupillary reflexes as indicated in this figure. This flow diagram shows the stratified results for treatment with and without LTx in terms of survival and neurological complications of each group. HE, hepatic encephalopathy; LTx, liver transplantation; PALF, pediatric acute liver failure.

To assess the diagnostic value of the *presence* of pupillary reflexes in patients with PALF grade III–IV, we first determined the outcome in terms of survival and neurological complications (Figure 1). Stratified results for treatment by LTx (*n* = 22) or without LTx (*n* = 15) showed an overall survival rate of 77% (*n* = 17/22) in good neurological condition. One patient survived with neurological impairment (cognitive impairment, requiring special education). After LTx, 5/22 children died, with 4/5 children due to postoperative complications and circulatory insufficiency and 1/5 child due to status epilepticus because of POLG disease (which had been diagnosed after transplantation). In the subgroup of children that did not undergo LTx, 9/15 children survived, of which 8/9 children were in a good condition and 1/9 child had neurological impairment (including a hemiparesis and a myoclonic movement disorder). Six of the 15 patients that did not undergo transplantation died, either due to multiorgan failure (*n* = 4) or due to intracerebral hemorrhage (*n* = 2).

#### 3.1.2 PALF Grade III‐IV Patients With Pupillary Reflexes Absent

To assess the diagnostic value of the absence of pupillary reflexes in patients with PALF grade III–IV, we first determined the outcome in terms of survival and neurological complications (Figure 1). In 10/14 patients with absent pupillary reflexes, cerebral imaging was performed. In 3/10 patients, imaging showed no indications for brain herniation, whereas in 7/10 patients, imaging revealed either uncal (6/7) or central transtentorial herniation (1/7). In the three patients with absent brain herniation, the PR had at least been absent for several hours (exact duration unknown). Absent PR in 2/3 patients appeared reversible after administration of hypertonic saline before the LTx. All 3/3 patients with absent PR and absent radiological signs of brain herniation survived in a good neurological condition (no neurological impairment). Three of the 7 patients with present radiological signs of brain herniation were transplanted within a median period of 11 (range 5–24) hours after the first radiological signs of brain herniation. In the three patients with brain herniation undergoing LTx, neurologic outcome was severe in terms of death (*n* = 2/3) or survival in a persistent minimally conscious state (*n* = 1/3). The remaining 4/7 children who did not receive a donor liver died from brain herniation. Table 3 shows the association between absent pupillary reflexes, radiological findings of brain herniation, and neurological outcome.

**TABLE 3:** Neurological outcome in patients with absent pupillary reflexes.

Case number	PR	OCR	CR	Brain herniation	LTx	Outcome
12	−/−	−/−	−/−	+; uncal	—	Dead
13	−/−	−/−	−/−	+; uncal	—	Dead
14	−/−	−/−	−/−	+; uncal	—	Dead
18	−/−	−/−	−/−	+; transtentorial	—	Dead
20	−/−	+/+	+/+	+; uncal	Yes	Dead
21	−/−	+/+	+/+	+; uncal	Yes	Dead
22	−/−	−/−	−/−	No	Yes	Alive
23	−/−	−/−	−/−	No	Yes	Alive
24	−/−	+/+	+/+	No	Yes	Alive

*Note:* Neurological outcome of PALF patients with absent pupillary reflexes and/or brain stem reflexes. In the absence of brain herniation, there was no relationship between outcome and absence of pupillary reflexes.

Abbreviations: CR, cornea reflex; LTx, liver transplantation; MSC, persistent minimally conscious state; OCR, oculocephalic reflex; PR, pupillary reflex.

Finally, there was a group of 4/14 patients with absent PR, in whom brain imaging was not performed. In these children, the severe clinical condition prohibited transportation to the CT or MRI before a donor organ became available. All patients died after multiorgan failure.

#### 3.1.3 Additional Value of Brain Stem Reflexes in Patients With or Without Pupillary Reflexes

In the patient group with present pupillary reflexes (*n* = 37), there were 10 patients with either uni‐ or bilateral *absence* of BSR (i.e., absence of OCR and/or CR). In this group, presence or absence of BSR was not related to the outcome in terms of survival or death (*p* = 0.232), Table 4. In the patient group with absent pupillary reflexes, 4/10 patients had bilateral *presence* of both OCR and CR, but this also was not significantly associated with outcome in terms of death (*p* = 0.166).

**TABLE 4:** Neurological outcome in patients with present pupillary reflexes.

Case number	PR	OCR	CR	LTx	Outcome
1	+/+	+/+	+/+	Yes	Dead
2	+/+	+/+	+/+	Yes	Dead
3	+/+	−/−	+/+	Yes	Dead
4	+/+	+/+	+/+	Yes	Dead
5	+/+	+/+	+/+	Yes	Dead
6	+/+	+/+	−/−	No	Dead
7	+/+	+/+	+/+	No	Dead
8	+/+	+/+	+/+	No	Dead
9	+/+	+/+	+/+	No	Dead
10	+/+	+/+	+/+	No	Dead
11	+/+	+/+	+/+	No	Dead
26	+/+	+/+	−/−	No	Alive
27	+/+	−/−	−/−	No	Alive
28	+/+	−/−	−/−	Yes	Alive
29	+/+	−/−	−/+	Yes	Alive
30	+/+	−/−	+/+	No	Alive
31	+/+	−/−	+/+	No	Alive
32	+/+	−/−	+/+	No	Alive
33	+/+	+/+	+/+	Yes	Alive
34	+/+	+/+	+/+	Yes	Alive
35	+/+	+/+	+/+	Yes	Alive
36	+/+	+/+	+/+	Yes	Alive
37	+/+	+/+	+/+	Yes	Alive
38	+/+	+/+	+/+	Yes	Alive
39	+/+	+/+	+/+	Yes	Alive
40	+/+	+/+	+/+	Yes	Alive
41	+/+	+/+	+/+	Yes	Alive
42	+/+	+/+	+/+	Yes	Alive
43	+/+	+/+	+/+	Yes	Alive
44	+/+	+/+	+/+	Yes	Alive
45	+/+	+/+	+/+	Yes	Alive
46	+/+	+/+	+/+	Yes	Alive
47	+/+	+/+	+/+	No	Alive
48	+/+	+/+	+/+	No	Alive
49	+/+	+/+	+/+	No	Alive
50	+/+	+/+	+/+	Yes	Alive
51	+/+	+/+	+/+	No	Alive

*Note:* Neurological outcome of patients with present pupillary reflexes and/or brain stem reflexes.

Abbreviations: −, absent; +, present; BMF, bone marrow failure; CR, cornea reflex; IB, intestinal bleeding; LTx, liver transplantation; n/a, not applicable; OCR, oculocephalic reflex; PR, pupillary reflex.

## 4 Discussion

In a Dutch nationwide observational cohort of patients with PALF and HE grade III‐IV, we retrospectively investigated the association between PR, radiological signs of brain herniation, and subsequent neurological outcome over three decades. We describe several children with absent pupillary reflexes who still had a good outcome, all after LTx. In contrast to the absence of pupillary reflexes, we did not observe a good outcome in any patient with radiological signs of brain herniation. Rather, all patients with radiological signs of brain herniation died or ended in a minimally conscious state (MCS), irrespective of whether they had been transplanted or not.

Our cohort consisted of 51 patients with PALF and HE grade III‐IV, with an overall survival of 59%. The best survival was found in patients with PR present, independent of the presence or absence of BSR. The survival rate from our cohort aligns with previously reported data from other large liver transplantation centers (PALF study group) [21].

Absence of pupillary reflexes to light is generally considered to be a parameter for brain herniation [22, 23, 24]. Our analyses, however, indicate that this interpretation is not uniformly justified. PR may become affected by other means than brain herniation, for example, by neurotoxicity, early reversible edema before brain herniation sets in, and weak, hardly discernible PR may be mistakenly interpreted as negative. The exact biochemical mechanism underlying neurotoxicity in patients with PALF and HE is still not fully understood, but the accumulation of neurotoxins, in particular ammonia, is likely to play a crucial role. This may result in an osmotic gradient with water influx into the astrocytes, causing cerebral edema. Administration of sedative medication is likely to enhance the existent neurotoxic response to ammonia [24, 25, 26], potentially influencing pupillary reflexes [24, 27, 28, 29]. In a proportion of children without brain herniation, this could theoretically also explain the good clinical outcome after LTx, despite absent PR and BSR prior to the transplantation. When PR are absent, clinical brain imaging is thus an important diagnostic tool for LTx treatment decisions.

In the presence of radiological brain herniation, our results indicate an invariably poor prognosis: no survival or survival in a minimally conscious state. These children were either diagnosed with uncal herniation (*n* = 6/7) and/or central transtentorial herniation (*n* = 1/7), in the absence of hemorrhage. Despite radiological herniation, three children in whom brain herniation had shortly evolved within hours while waiting for LTx were still transplanted in the hope that the process of herniation was still reversible. However, we observed in these children an irreversibly severe outcome, being either death (*n* = 6) or a minimally conscious state (*n* = 1). In the latter child, radiological brain herniation was reflected by an initiating white cerebellar sign on CT (consisting of decreased density of the supratentorial brain parenchyma, and increased density of the cerebellum) [30]. In the clinical literature, this sign is explained as imminent mortality due to co‐existent high intracranial pressure, cerebellar swelling, and herniation. However, this is not invariably true since instantaneous elimination of the intracranial pressure can still result in a favorable prognosis [22]. In our study, the patient with a white cerebellum was transplanted within hours; nevertheless, with an outcome of a persistent minimally conscious state.

In patients with preserved PR, we observed a good neurological prognosis in 92%, or mild neurological impairment in 8% of the children, independent of LTx or conservative treatment (77% survival after LTx and 60% after conservative treatment without LTx). Although this suggests that children may have a good prognosis as long as PR is still present, we realize that it is unpredictable whether the clinical condition will ultimately irreversibly deteriorate due to brain herniation. Based on the current work and to rule out the uncertainty of the timely availability of a deceased donor, we have introduced in our center a protocol for acute living donor liver transplantation for PALF [31]. With this protocol, we have dramatically shortened the pre‐LTx waiting time by screening living donors and performing the subsequent transplantation within 24 h after the decision to proceed to liver transplantation. We expect that this strategy will further improve survival rates and neurological outcomes for PALF [21, 32, 33, 34, 35].

Altogether, in addition to the evaluation of PR's, we did not find an additional prognostic value of the assessment BSR's in our cohort. Among the patients with present PR, we observed absence of OCR and/or CR in 27%. This can theoretically be attributed to the fact that the PR is one of the most resistant brain responses during metabolic encephalopathy [20]. Conversely, however, this cannot explain why some (*n* = 4/10) of the patients with absent PRs still revealed persistent BSR. Interpretation of pupil responses to light may vary due to differences in light intensity and duration of the light stimulus, whereas other factors such as medication, brain condition, age, and diabetes mellitus may confound the response. However, despite that, our results show that simple manual pupil assessment provides a valuable parameter to decide when additional brain imaging is needed.

We recognize some limitations of this study. First, we are aware of the (still) relatively small sample size of this study. However, pediatric acute liver failure is a rare condition, and this study is conducted in a relatively large national European center for liver transplantation including all, except one, eligible cases from the past 30 years. Second, we are also aware that we did not use an automatic pupillometer, which is suggested to be more precise in documenting PR with its own limitations. However, the automatic pupillometer has its own limitations such as missing very slowly reacting pupils. In clinical practice, a penlight is still considered to be accurate, frequently used, and easier to interpret than an automatic pupillometer [36, 37]. Third, we used the West Haven Criteria for clinical grading of HE rather than electroencephalography (EEG) outcome data to determine the grade of HE. Nonetheless, the West Haven Criteria is the most frequently used scale for grading the extent of HE and has also proved to be reliable [14]. Finally, in the present study, we deliberately confined ourselves to the major neurological outcome in terms of survival and/or major neurological impairment. In the future, we will investigate a more detailed overview of the neurological outcome and morbidity in the surviving children with PALF grade III‐IV.

In summary, neurological survival after PALF is favorable in the majority of patients with HE grades III–IV, provided radiological signs of brain herniation are absent. However, during pre‐LTx waiting time, the neurological prognosis may irreversibly deteriorate, eventually resulting in absent PR and radiologic signs of brain herniation. We therefore advocate that the absence of PR should be an indication for radiological imaging to assess brain herniation before major treatment decisions.

## Conflicts of Interest

The authors declare no conflicts of interest.

## Data Availability

We are unable to share our research data as it contains sensitive and confidential information.
